# Identification of DREB gene family in foxtail millet (*Setaria italica*) and analysis of its expression pattern in response to abiotic stress

**DOI:** 10.3389/fpls.2025.1552120

**Published:** 2025-04-28

**Authors:** Yanan Yang, Yun Li, Zhenqing Guo, Yuxue Zhao, Xiaoke Zhou, Yucui Han, Xiaohu Lin

**Affiliations:** ^1^ College of Agronomy and Biotechnology/Hebei Key Laboratory of Crop Stress Biology, Hebei Normal University of Science and Technology, Qinhuangdao, China; ^2^ Research Center of Rural Vitalization, Hebei Normal University of Science and Technology, Qinhuangdao, China

**Keywords:** foxtail millet, DREB, gene family, abiotic stress, gene expression

## Abstract

Dehydration response element binding proteins (DREBs) play a vital role in transcriptional regulators in enhancing plant tolerance to abiotic stress. To investigate the biological functions of the DREB gene family (*SiDREBs*) in foxtail millet (*Setaria italica*), this study performed bioinformatics and gene expression analysis on *SiDREBs* under abiotic stress. A total of 166 family members of *SiDREBs* were identified, which were classified into six subfamilies. *SiDREBs* were unevenly distributed on nine chromosomes, and were designated as *SiDREB1*–*166* based on their chromosomal positions. Covariance analysis revealed that *SiDREBs* were much more closely related to monocotyledonous plants sorghum, maize, and rice than to dicotyledonous plants *Arabidopsis thaliana*, tomato, and soybean. Promoter *cis*-acting element analysis showed that *SiDREBs* contained stress-related *cis-*acting elements. Under saline-alkali stress, *SiDREB153* exhibited significantly different expression levels between the resistant and susceptible materials, indicating that it plays a positive regulatory role in the response of foxtail millet to saline-alkali stress. Among different abiotic stresses, the expression of *SiDREB80* increased under drought, saline-alkali, and shade stress, that of *SiDREB4/129/131* rose under saline-alkali and high temperature stress, and that of *SiDREB159* increased under herbicide and saline-alkali stress. These genes play an important role in the response of foxtail millet to stress. These findings provide a theoretical basis for further studies on the function of *SiDREBs* in response to abiotic stress.

## 1 Introduction

Under global climate change, the growth and development of plants are challenged by increasingly serious abiotic stresses, which have posed great challenges to global agricultural production (Su et al., 2023). In response to all kinds of abiotic stresses, plants have evolved a suite of complex regulatory mechanisms in the process of evolution, in which stress response transcription factors and hormone signal transduction work together to form an interconnected regulatory network (Devireddy et al., 2021). According to domains, the AP2/ERF superfamily can be split into four subfamilies, namely *AP2*, *DREB*, *ERF* and *RAV* (Sakuma et al., 2002). Although the sequence of the AP2/ERF domain is highly conserved, the DNA binding characteristics of the four subfamilies are different (Wu et al., 2022).

The dehydration response element binding (DREB) family regulates abscisic acid (ABA) by activating stress response to independent of ABA pathways through dehydration-responsive elements (DREs). DREB is considered as one of the most important gene families involved in abiotic stress response, as ABA is essential for plant growth under stress conditions (Maqsood et al., 2022). For example, it plays an important regulatory role under drought, salt, high temperature and cold stress (Ain-Ali et al., 2021). According to the domain, *SiDREBs* are divided into A1, A2, A3, A4, A5 and A6 subfamilies. It has been reported that *DREB2A* and *DREB2B* play an essential role in response to drought, salt, and high temperature stresses in the A2 subfamily of Arabidopsis (Nakashima et al., 2000; Agarwal et al., 2006). Overexpression of *DREB3A* from *Leymus chinensis* improved the drought and salt tolerance of Arabidopsis (Xianjun et al., 2011). Functional analysis of the *DREB2* gene in *Broussonetia papyrifera* showed that it is involved in drought and salt stress response (Mushtaq et al., 2021). These results indicate that the DREB gene family members contribute to resistance to abiotic stress. DREB is a polygenic family with a single conserved AP2 domain (Dietz et al., 2010). This domain is related to the defense mechanism of plants against external environmental pressure (Dietz et al., 2010). The DREB genes bind to the drought stress responsive element (DRE/CRT) under the action of seven amino acids (including one V residue, four R residues, and two W residues) (Allen et al., 1998; Mushtaq et al., 2021). Under abiotic stress, the core motif of DRE/CRT can directly interact with *DREB* genes (Vazquez-Hernandez et al., 2017). Currently, the DREB gene family member have been identified in many plants, such as *Arabidopsis thaliana* (Hwang et al., 2012), *Pennisetum glaucum*, *Fragaria ananassa*, *Nicotiana tabacum* (Agarwal et al., 2007), *Oryza sativa*, *Lactuca sativa* (Park et al., 2020), *Musa paradisiaca*, *Triticum aestivum*, *Capsicum annuum*, *Zea mays* (Liu et al., 2013), *Glycine max* (Hou et al., 2022), and *Solanum tuberosum* (Mushtaq et al., 2021).

Foxtail millet (*Setaria italica*) is one of the main food crops in northern China, as well as a typical C4 plant with the characteristics of drought and salt tolerance. It has the characteristics of environmental protection (Wang et al., 2024). Foxtail millet was domesticated from *Setaria viridis*, and is a genus belonging to the Gramineae family and is closely related to many other crops, such as *Zea mays*, *sorghum bicolor*, *Pennisetum glaucum*, *Panicum virgatum*, *Pennisetum purpureum*, and *Saccharum officinarum* (Doust et al., 2009). Foxtail millet has a small genome and a short life cycle (Muthamilarasan and Prasad, 2015), and is a good model crop for studying stress response and gene function (Bennetzen et al., 2012). In this study, we carried out a comprehensive bioinformatics analysis of *SiDREBs* in terms of gene family member identification, chromosome location, gene structure, phylogeny, gene tissue expression specificity, and abiotic stress response, providing a gist for further gene function study.

## 2 Materials and methods

### 2.1 Test materials and saline-alkali stress treatment

In this study, the salt-tolerant foxtail millet variety JK3 and the salt-sensitive B175 screened by our research group were used as experimental materials. Foxtail millet seedlings were grown in incubators. After NaCLO disinfection, the seeds were flushed with distilled water, soaked in distilled water and cultured for 24 h, sown, and placed in artificial climate tank (Day/night duration: 12h/12h; Day/night temperature: 28°C/22°C; Humidity: 65%). The seedlings were cultured to three leaves and one heart, and treated with 75% artificial seawater (the salinity of the original seawater is 2.7%.) taken from the Bohai Sea area of Qinhuangdao Port, Hebei Province for saline-alkali stress. The same volume of distilled water was used as the control treatment. The second and third leaves of millet treated for 0 h, 12 h and 24 h were sampled, immediately frozen in liquid nitrogen, and stored at –80°C for RNA extraction. Each treatment had three biological replicates.

### 2.2 Identification of *SiDREB* family members and analysis of their protein physicochemical properties

The fasta. and Gff3. files of *Setaria italica* were downloaded from the Phytozome database(Phytozome (doe.gov). The protein sequence of Arabidopsis DREB gene family was downloaded from NCBI database(National Center for Biotechnology Information (nih.gov). The candidate genes of *SiDREBs* were screened by blast comparison with the total protein sequence of foxtail millet using TBtools software. Also, the hmm. file (PF00847) was downloaded from the Pfam database (http://pfam.xfam.org/). The candidate genes identified by the two methods were intersected to screen the members of the *SiDREB* family. The SMART database (http://smart.embl-heidelcbi.nlm.ni and InterPro database (InterPro (ebi.ac.uk)) were used to validate and eliminate incomplete conserved domain sequences. Finally, *SiDREBs* were determined.

The physicochemical properties of *SiDREBs* by Expasy (ProtParam-SIB Swiss Institute of Bioinformatics| Expasy). SignaIP 4.1 Server (http://www.cbs.dtu.dk/services/SignalP-4.1/)) was used to analyze the signal peptide of DREB protein. The transmembrane region of DREB protein was analyzed by TMHMM (http://www.cbs.etu.dk//cgi-bin/). The subcellular localization of DREB protein sequences was predicted by WoLF PSORT (https://wolfpsort.hgc.jp/).

### 2.3 Phylogenetic tree construction, gene structure and conserved motif analysis of *SiDREBs*

Protein sequences of DREB gene family in Arabidopsis and rice were downloaded from NCBI database. MEGA11 was used to compare 166 DREB protein sequences of foxtail millet, 57 DREB protein sequences of rice and 57 DREB protein sequences of Arabidopsis. Neighbor-joining method (NJ) was used to construct the phylogenetic tree. Landscaping of the phylogenetic tree was completed using the online tool ITOL (https://itol.embl.de/)). They were designated as *SiDREB1*~*166* according to their chromosomal positions.

The gene structure, conserved domains, and conserved motifs of SiDREBs were analyzed by TBtools and the results were visualized.

### 2.4 Chromosomal localization, gene duplication, and collinearity analysis of *SiDREBs*

According to the annotation information of foxtail millet genome, the location information of the chromosomes of *SiDREB* family members was obtained. To learn more about *SiDREBs* evolution, we selected the dicotyledonous representative species Arabidopsis, soybean, and tomato, and the monocotyledonous representative species rice, maize, and sorghum to align with the genome sequence of foxtail millet, respectively. Collinear relationships between millet and these species were obtained, and the collinearity map between species was drawn by TBtools. Finally, DNA SP V5.0 was used to calculate and analyze nonsynonymous (Ka) and synonymous substitution (Ks), and the selection pressure was analyzed according to the value of Ka/Ks. The genome and annotation information of all species except Arabidopsis was obtained from the Phytozome database.

### 2.5 Prediction of *cis*-acting elements in the promoter region of *SiDREBs*

The 2000-bp upstream sequence of the identified *SiDREBs* was submitted to the PlantCARE (http://bioinfor-matics.psb.ugent.be/webtools/plantcare/html/) website to analyze the *cis*-acting elements in the promoter region.

### 2.6 GO and KEGG enrichment analysis of *SiDREBs*

Based on the previous transcriptome data of the research group, GO and KEGG files were analyzed using the OUYI platform ((oebiotech.com).). Get gene function annotation file.

### 2.7 Analysis of the expression patterns of *SiDREBs* in different tissues

The expression data of *SiDREBs* in different tissues at different stages were obtained from Phytozome database and submitted to TBtools software to analyze and draw the tissue-specific expression heat map of DREB family genes.

### 2.8 Interaction network analysis of SiDREBs

The protein interaction network was constructed by STRING. (https://string-db.org/). The maximum number of interactors was set to five to predict the protein function of *SiDREBs*. Secondary and tertiary structures were predicted using SOPMA (http://npsa-pbil.ibcp.fr/cgibin/npsa_automat.pl?page=npsa_sopma.html) and SWISS-MODEL (https://swissmodel.expasy.org/).

### 2.9 Expression analysis of *SiDREBs* under different abiotic stresses

Based on previous transcriptome data of foxtail millet under saline-alkali stress at 0, 12 and 24 h, the absolute value of Log2 FC greater than 1 was used as the standard (Zhang et al., 2023), and the differentially expressed genes (DEGs) were screened. The transcriptome sequencing data of millet leaves under high temperature stress (PRJNA756390) (Huang et al., 2021), drought stress (PRJEB21225) (Tang et al., 2017), shade stress (PRJNA772942) (Liu et al., 2022), and herbicide stress (PRJNA751769) (Sun et al., 2022) were retrieved and downloaded from the NCBI database. The plugin of TBtools was used to obtain the gene expression quantity and to construct the expression heat map of *SiDREBs* in abiotic stress response.

## 3 Results

### 3.1 Member identification and physicochemical property analysis of *SiDREBs*

By comparing the DREB protein sequences of Arabidopsis and foxtail millet with Blast, combined with the results of HMM identification, the conserved domain identification of the obtained *SiDREBs* was carried out, and finally 166 *SiDREBs* were determined (**Supplementary Table S1**). According to the chromosomal positions of *SiDREBs*, they were re-named as *SiDREB1*–*166*. The physicochemical properties of SiDREB proteins were analyzed. The amino acid (aa) length was 83 aa (SiDREB136) to 698 aa (SiDREB102), and the molecular weight was 9513.65–72864.45 Da. The PI of SiDREBs ranged from 4.45 (SiDREB121) to 11.72 (SiDREB116), and the PI of most SiDREBs was lower than 7, indicating that most SiDREBs are rich in acidic amino acids and are acidic proteins. The instability index of SiDREBs ranged from 34.62 (SiDREB149) to 86.46 (SiDREB72), and only a small number of members had an instability index lower than 40, indicating that the proteins are mostly unstable proteins. The lipid solubility index (AI) ranged from 45.95 (SiDREB7) to 86.46 (SiDREB72). Prediction of the hydrophilicity and hydrophobicity revealed that only one member, SiDREB115, had a hydrophilicity coefficient (GRAVY) greater than 0, indicating that it is a hydrophobic protein, while the remaining proteins are hydrophilic. The subcellular localization results showed that 112 SiDREBs were located in the nucleus, and the remaining members were located in chloroplast (43), cytoplasm (9), mitochondria (9), vacuole (1) and extracellular matrix (1). Prediction of signal peptide and transmembrane region showed that SiDREBs had no signal peptide, while only SiDREB97 and SiDREB130 had the transmembrane region. These results suggested that the family proteins could not guide the transmembrane transport of proteins.

### 3.2 Phylogenetic tree, gene structure, and conserved motif analysis of *SiDREBs*

**Figure 1:**
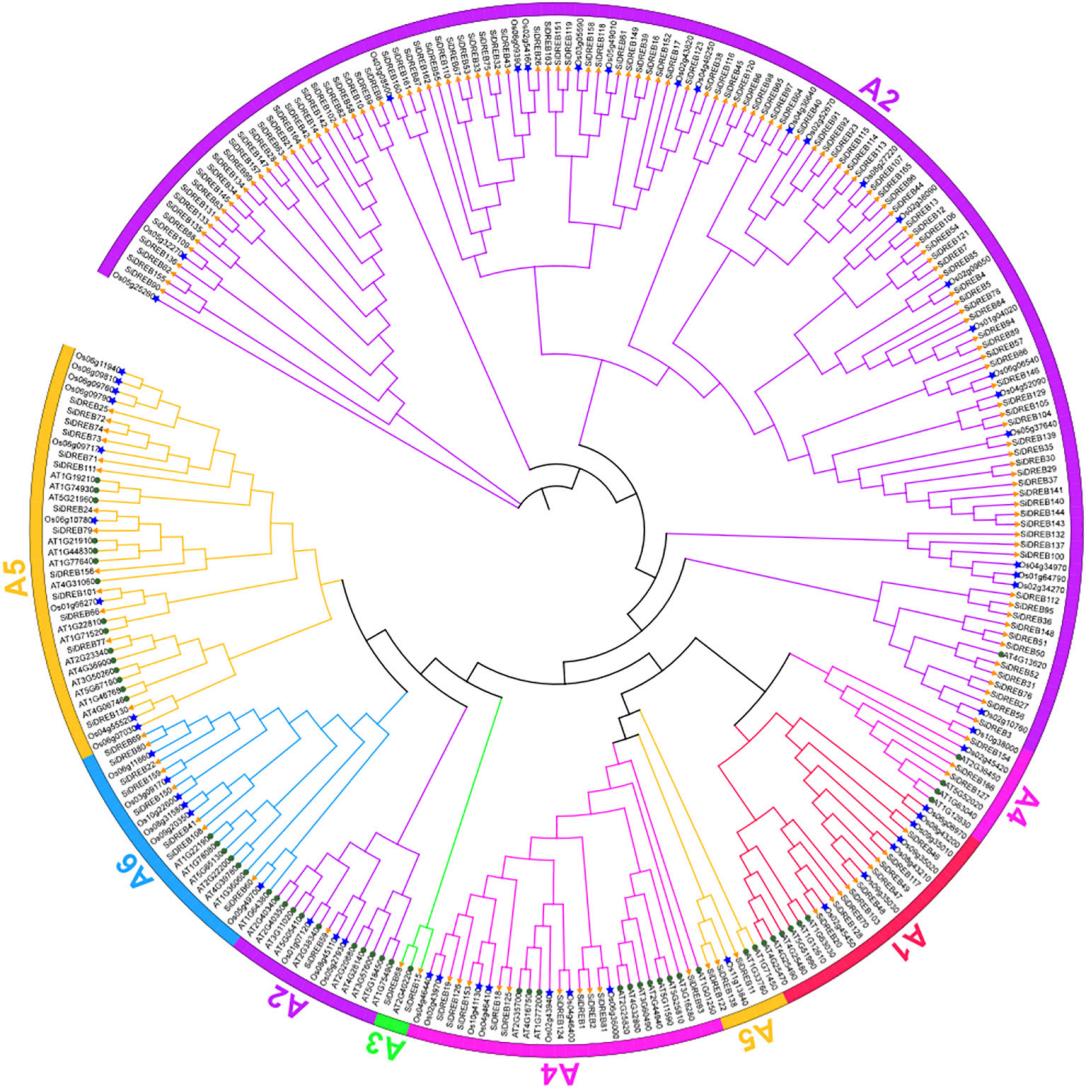
Phylogenetic tree of DREB gene family in foxtail millet, rice and Arabidopsis. There are 57 *AtDREBs.* (circles), 57 *OsDREBs* (asterisk), and 166 *SiDREBs* (triangle). The different colored branches represent different subfamilies.

In order to analyze the evolutionary pattern of *SiDREBs*, the 166 protein sequences of SiDREBs identified in this study were combined with the DREB protein sequences of Arabidopsis (57) and rice (57) to construct phylogenetic trees (**Figure 1**). *SiDREBs* were separated into six subfamilies (A1–A6). A2 was the largest subfamily with 118 members, and A3 was the smallest subfamily with only two members. These results indicated that various subfamilies might perform distinct roles.

Tbtools was used to predict the conserved domain, conserved motifs, and gene structure of *SiDREBs*, and 10 conserved motifs of *SiDREBs* were identified by MEME (**Supplementary Figure S1A**). The quantity of motifs varied throughout subfamilies, and the motifs of members in the same subfamily were similar. Among them, *SiDREB69* did not contain motif 1, while *SiDREB136* had no motif 2, and the remaining genes all contained motif 1 and motif 2, indicating that *SiDREBs* are highly conserved. Motif 3 was located in the N-terminal DREB domain, while motif 4 and motif 9 were located in the C-terminal DREB domain. Most *SiDREBs* in a given subfamily had a very similar arrangement and number of conserved motifs, indicating that these genes were conserved during evolution with relatively conserved functions.

The structure of *SiDREBs* gene was further analyzed. The results showed that most of the *SIDREBs* genes were broken genes (**Supplementary Figure S1B**). Most *SiDREB* genes had no introns, but the number of introns in the A2 subfamily could reach up to 9, which is similar to the distribution ratio of exons. Members of different subfamilies showed diverse numbers and locations of exons and introns. Members of the same subfamily had relatively conserved location of exons and introns, that could be caused by the members of the same subfamily’s tight evolutionary relationships.

### 3.3 Chromosome localization, gene replication, and collinearity analysis of *SiDREBs*

**Figure 2:**
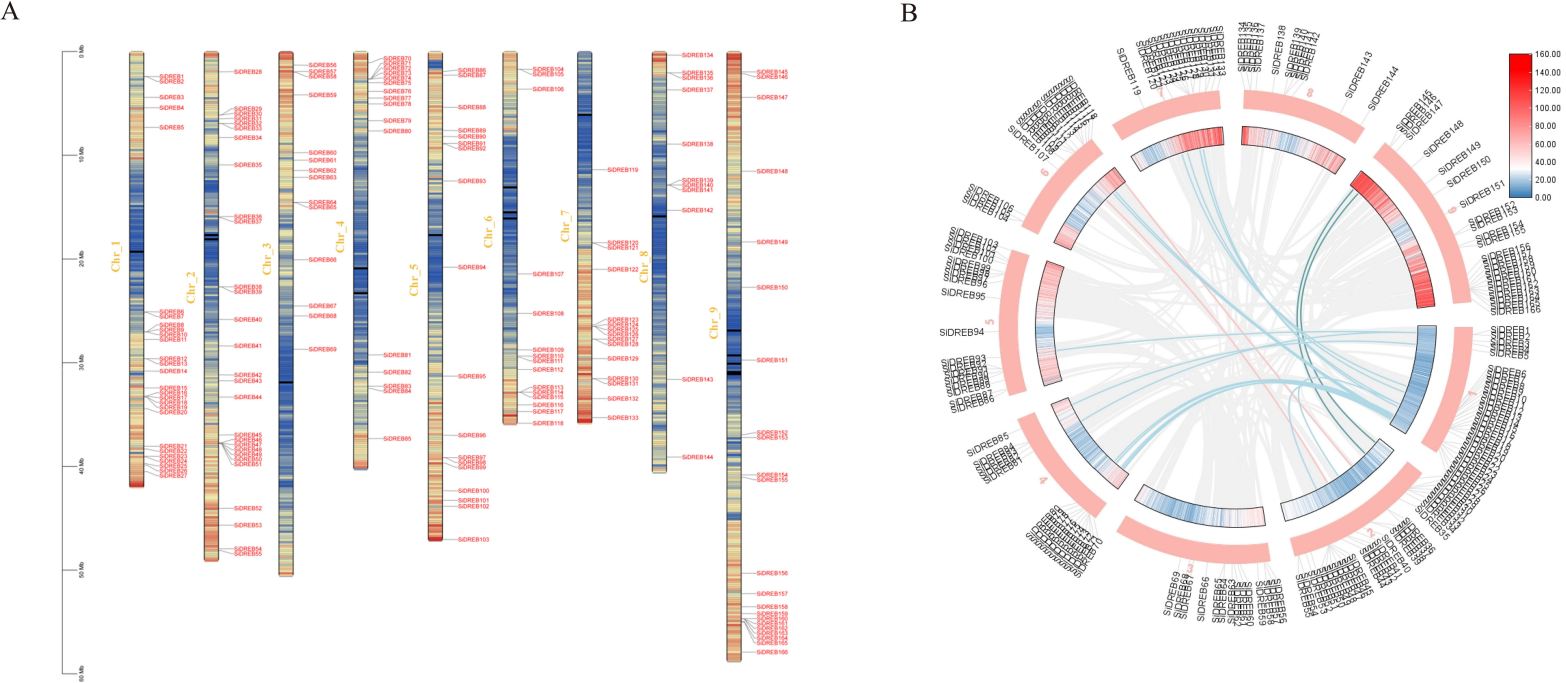
Chromosome localization and inter-chromosomal synthetic region analysis of *SiDREBs*. **(A)** The various colors of the inner ring represent the gene density of the chromosome (from blue to red), **(B)** The blue, pink and green lines represent the repeated gene pairs of foxtail millet.

The chromosomal localization of *SiDREBs* was determined by using TBtools tool mapping (**Figure 2A**). The 166 *SiDREBs* were unevenly distributed on nine chromosomes, among which chromosome 1 had the greatest number of genes (27 genes), and chromosome 8 had the fewest genes (11 genes). In addition, 21 pairs of homologous genes were identified to be involved in fragment duplication, accounting for 23% of the total. The results suggested that these genes may be formed by replicating large segments of chromosomes (**Figure 2B**).

**Figure 3:**
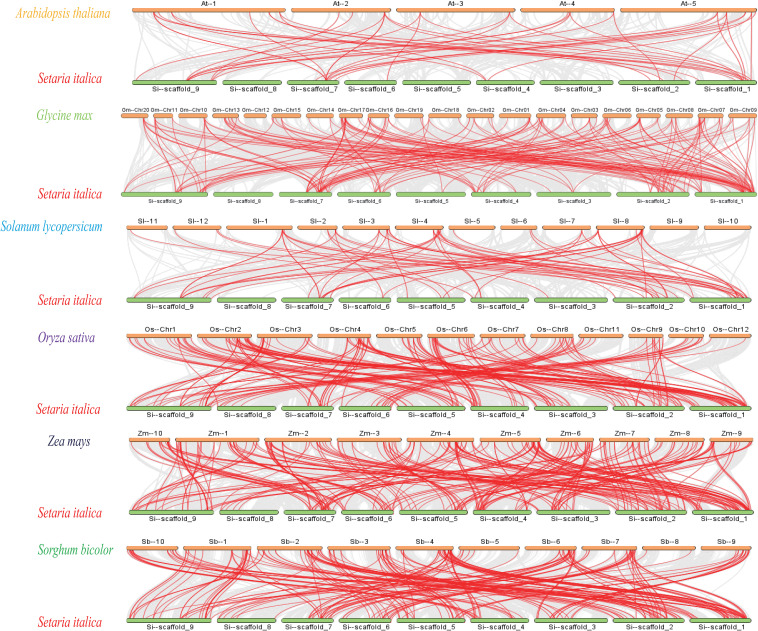
Analysis of DREB genes among foxtail millet, Arabidopsis, tomato, sorghum, soybean, rice and maize. Gray lines represent the collinear relationship between foxtail millet and six other species and red lines represent collinear DREB gene pairs.

In order to further study the phylogenetic mechanism of *SiDREBs*, we analyzed the collinear diagram of foxtail millet and six representative species (three dicots: Arabidopsis, soybean and tomato; three monocots: rice, maize and sorghum) (**Figure 3**). In terms of homologous genes of *SiDREBs* with other species, sorghum had the most homologous genes (130 pairs), followed by rice (129 pairs), maize (124 pairs), soybean (100 pairs), tomato (38 pairs), and Arabidopsis (24 pairs). There was no collinear gene between foxtail millet chromosome 8 and dicotyledonous plants. In addition, *SiDREB80/120/127/128/153/159* had homologous genes with these six plants. These results indicated that these genes might have existed earlier to the differentiation of plants. To better understand the evolutionary process of *SiDREBs*, the Ka, Ks, and Ka/Ks values of the repeated gene pairs were calculated (**Supplementary Table S2**). The results demonstrated that the Ka/Ks ratios of all gene pairs ranged from 0.2 to 0.9, which were all below 1, indicating that the evolution of *SiDREBs* was affected by strong purification selection, and the gene function tends to be conserved.

### 3.4 Analysis of *cis*-acting elements in the promoter region of *SiDREBs*

**Figure 4:**
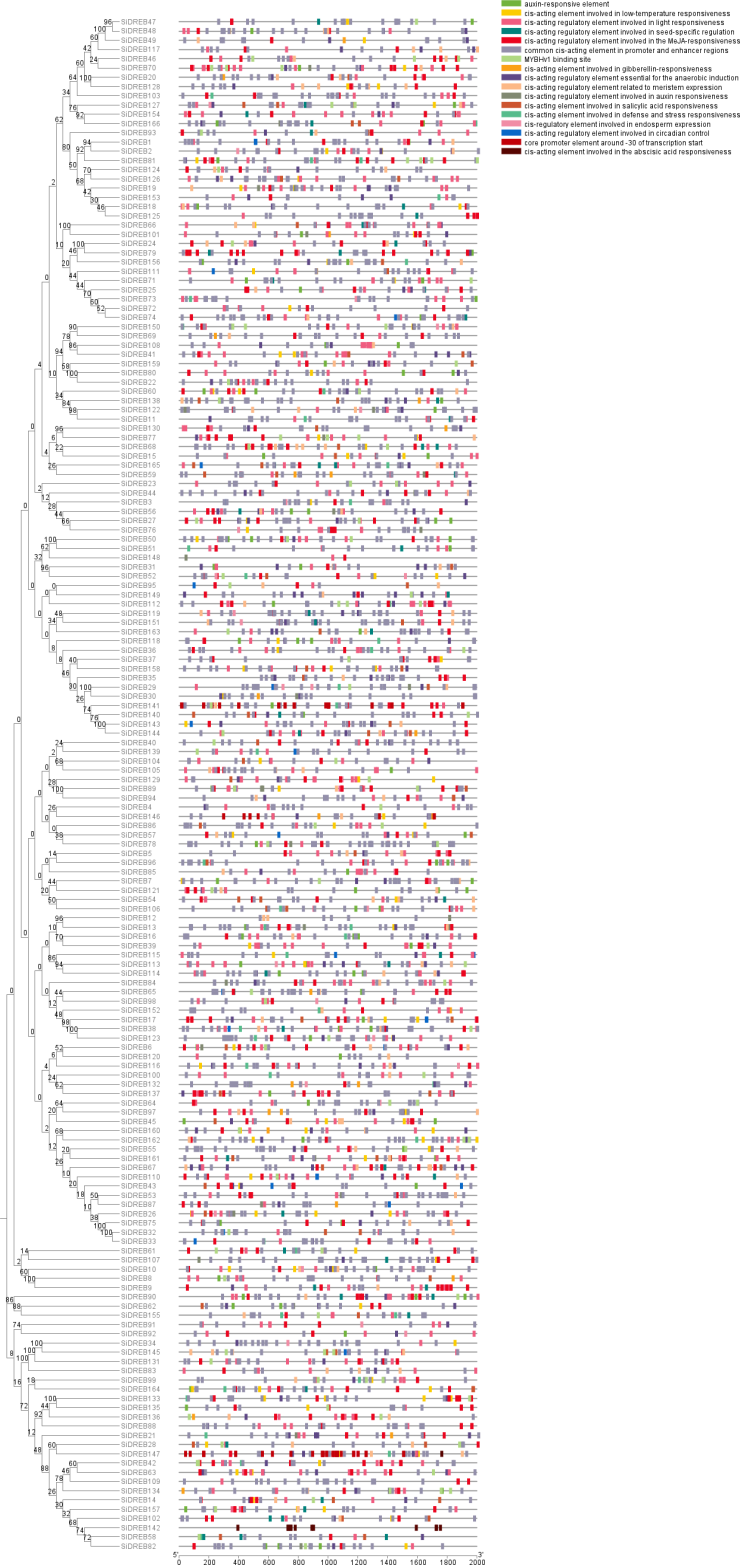
*Cis*-acting element analysis of *SiDREB* promoter regions. Various colored blocks stand for various *cis*-acting elements.

In order to study the regulation of *SiDREBs*, we forecasted and analyzed the *cis*-acting elements in their promoter region (**Figure 4**). The *cis*-acting elements of the family members could be mainly split into four categories in the light of the functional annotation. The first category included light-responsive elements; the second category comprised hormone-responsive elements such as auxin and gibberellin. The third category included stress-responsive elements, such as low temperature responsive elements. The fourth category included *cis*-acting elements related to growth and development, such as meristem or endosperm expression. Different *SiDREB* members contained different types of *cis*-acting elements, but all members had light-responsive elements. Among all *SiDREBs*, 77 members contained low temperature responsive elements and 42 members had defense and stress responsive elements; suggesting that *SiDREBs* could be crucial for the response to abiotic stress. *SiDREB79* and *SiDREB70* harbored the most stress responsive elements, indicating that these two genes may play an important regulatory role in plant stress response.

### 3.5 Go and KEGG enrichment analysis of *SiDREBs*

**Figure 5:**
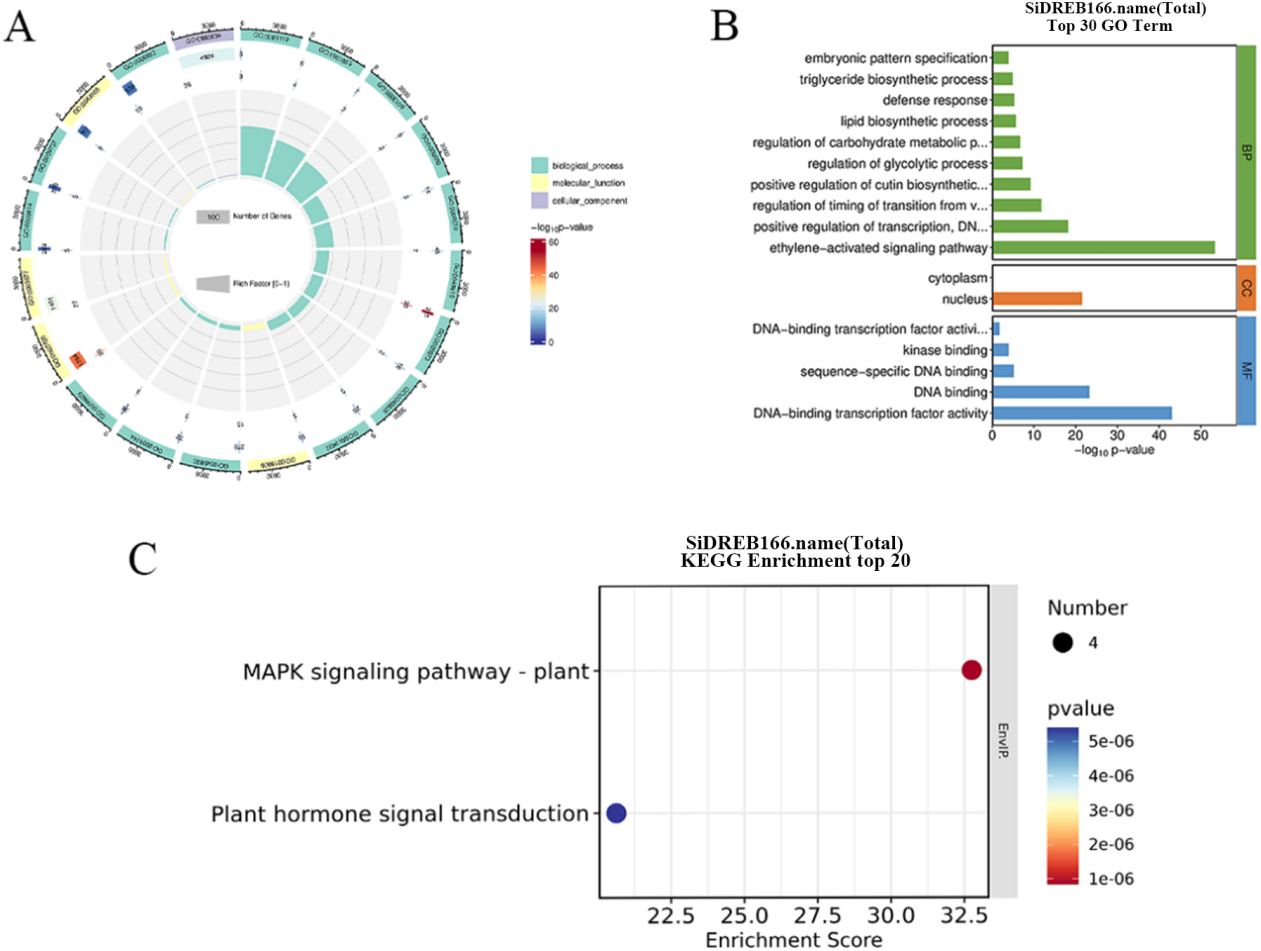
GO and KEGG enrichment analysis of *SiDREBs*. **(A)** Circus plot of GO enrichment analysis of 166 *SiDREBs* (all GO terms). **(B)** Histoplot of GO enrichment analysis of 166 *SiDREB* genes (top 30 enrichment items). **(C)** KEGG enrichment analysis scatter plot of 166 *SiDREBs* (top 20 enrichment terms).

The GO enrichment analysis of *SiDREB* genes showed that 47, 2 and 5 GO entries were enriched in biological processes, cellular components, and molecular functions, respectively (**Figure 5A**). In terms of biological processes, *SiDREBs* were mainly enriched in ethylene-activated signaling pathways and positive regulation of DNA-templated transcription. In terms of cell components, *SiDREBs* were mainly enriched in nucleus. In terms of molecular functions, *SiDREBs* were mainly enriched in DNA-binding transcription factor activity, and DNA binding. (**Figure 5B**).

KEGG pathway enrichment analysis of *SiDREBs* showed that four genes (*SiDREB37/139/141/158*) were significantly enriched in two pathways, including the MAPK signaling pathway and hormone signal transduction pathway (**Figure 5C**).

### 3.6 Expression analysis of *SiDREBs* in different tissues

**Figure 6:**
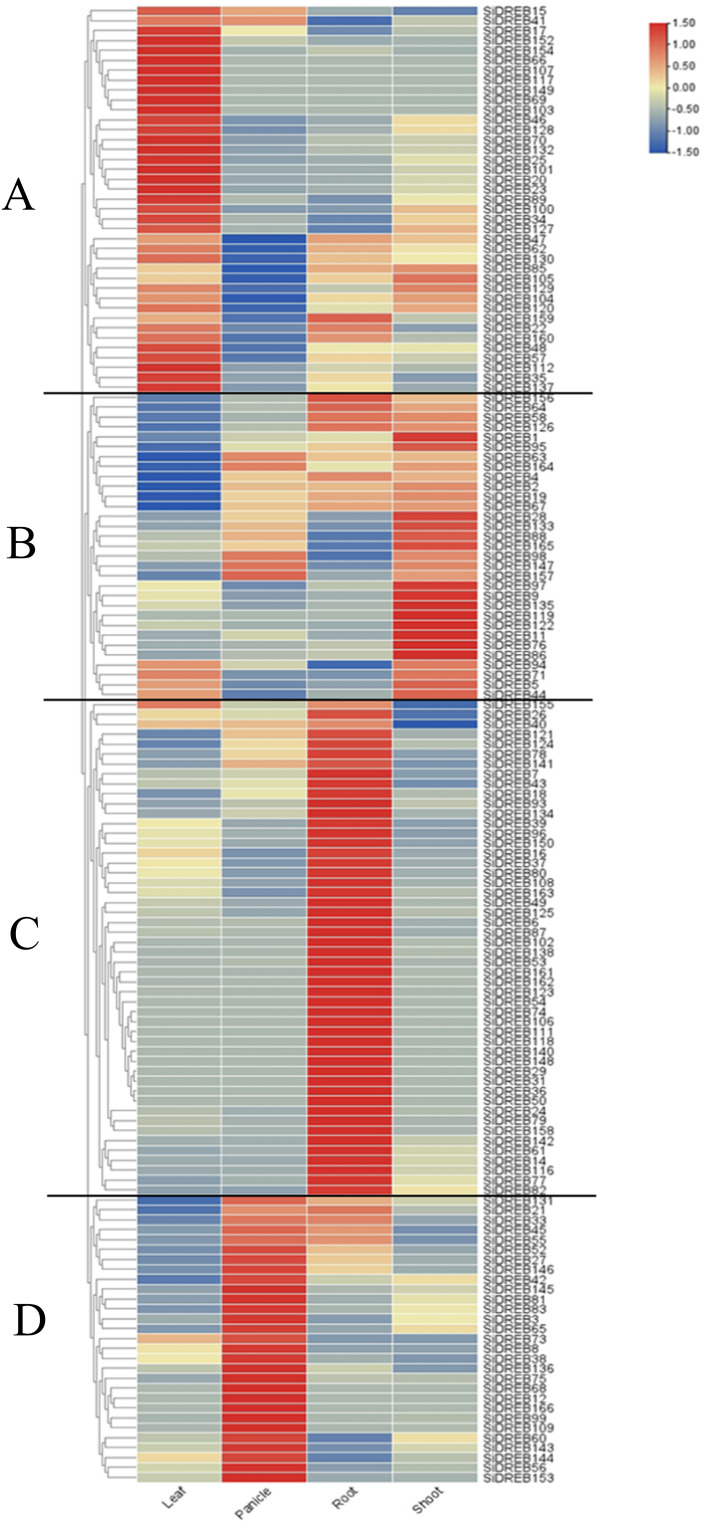
Tissue expression profiles of *SiDREBs*. The expression patterns of 166 *SiDREBs* in leaves, panicles, roots and shoots of foxtail millet. *SiDREBs* were divided into four branches (**A–D** represents the first, second, third and fourth branches respectively). The blocks that are blue and red stand for down and up, respectively.

To further understand the expression pattern of DREB, the expression profiles of *SiDREBs* in leaves, roots, shoots, and panicles were constructed by using the RNA-seq data of foxtail millet in Phytozome database. According to cluster analysis, *SiDREBs* were divided into four main branches, and most of the genes were highly expressed in leaves, shoots, roots, and panicles, respectively. The genes of the first branch were highly expressed in leaves (**Figure 6A**). Most of the genes in the second branch were highly expressed in roots, shoots, and panicles, suggesting that the growth and development of foxtail millet are significantly influenced by these genes (**Figure 6B**). The genes of the third branch were highly expressed in roots and lowly expressed in shoots, indicating that these genes have strong specificity (**Figure 6C**). The genes of the fourth branch were highly expressed in the panicle, indicating that the development of panicles depends critically on these genes (**Figure 6D**).

### 3.7 Prediction of SiDREB-interacting proteins and analysis of protein secondary and tertiary structure

**Figure 7:**
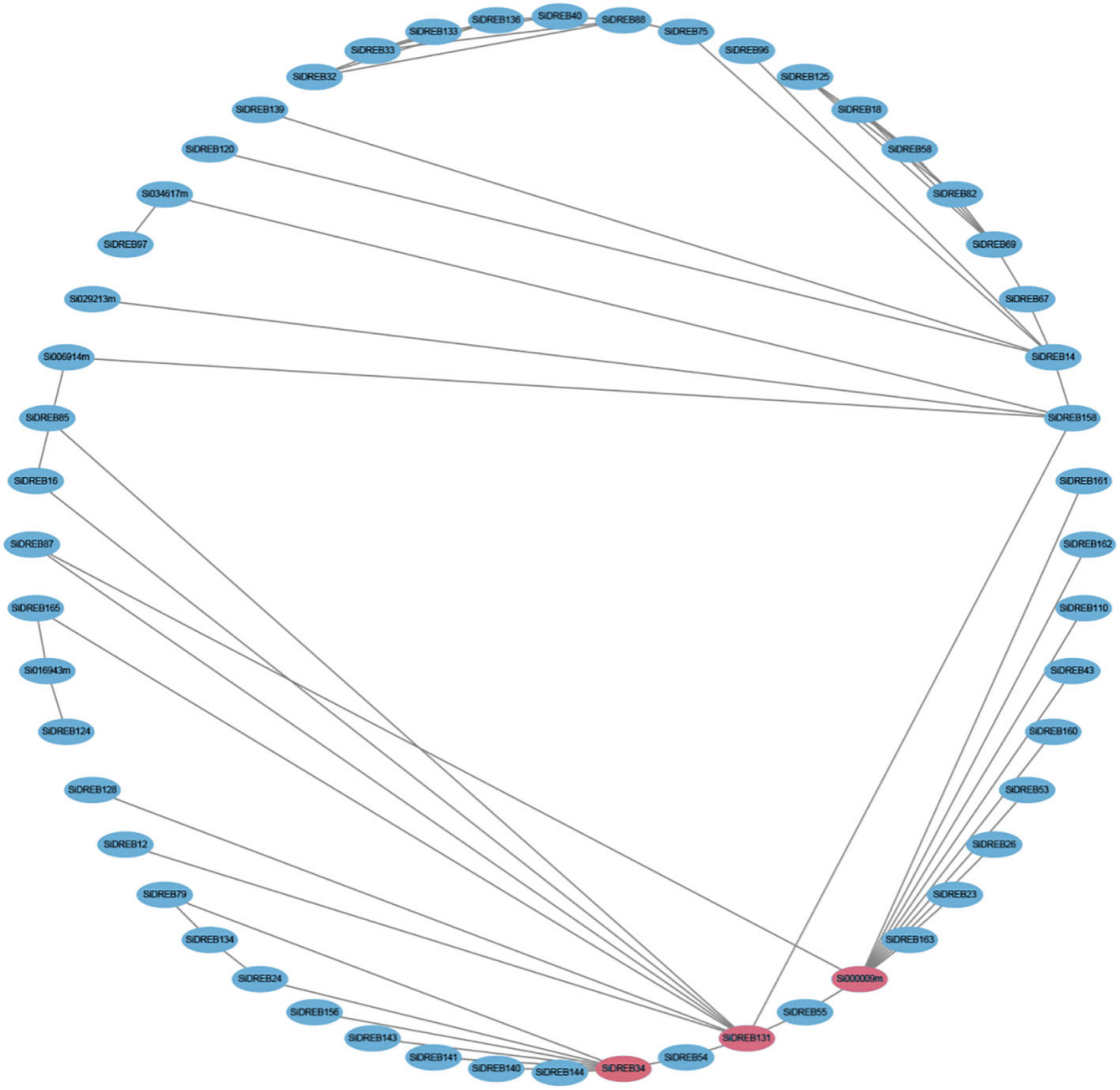
Prediction network of protein interactions for SiDREBs. Each node represents a protein, and each edge represents an interaction. The protein with the most frequent interactions is highlighted with red.

Protein-protein interaction network can help predict functional orthologous proteins in sequence homology clusters, which has important significance for studying gene interaction and regulatory relationships. Therefore, we used the STRING online database to analyze the protein interactions of SiDREBs (**Figure 7**). There were a large number of interactions between different DREB proteins in the whole regulatory network. SiDREB131 and SiDREB34 had nine and eight interacting proteins, respectively, suggesting that these two proteins may be core proteins of SiDREBs. We also found that Si000009m interacted with the largest number of proteins (11 SiDREBs proteins), all of which belonged to the A2 subfamily. Therefore, we speculate that the function of members in this subfamily may be highly correlated with each other.

The secondary structure of 47 DREB gene family members in foxtail millet was further analyzed by SOPMA online software (**Supplementary Figure S2**). The results showed that all the predicted family members had α-helix, random coil, and extended chain, but β-turn was not found. α-helix and random coil were dominant in the secondary structure of SiDREB proteins. SWISS-MODEL was further used to analyze the protein tertiary structure of these members (**Supplementary Figure S3**). The results showed that the tertiary structure of 47 SiDREBs proteins was mainly composed of random coils, and the spatial structure of 47 SiDREB proteins was highly similar. Therefore, it can be speculated that the functions of these proteins are similar and closely related.

### 3.8 Expression analysis of *SiDREBs* under different abiotic stress

**Figure 8:**
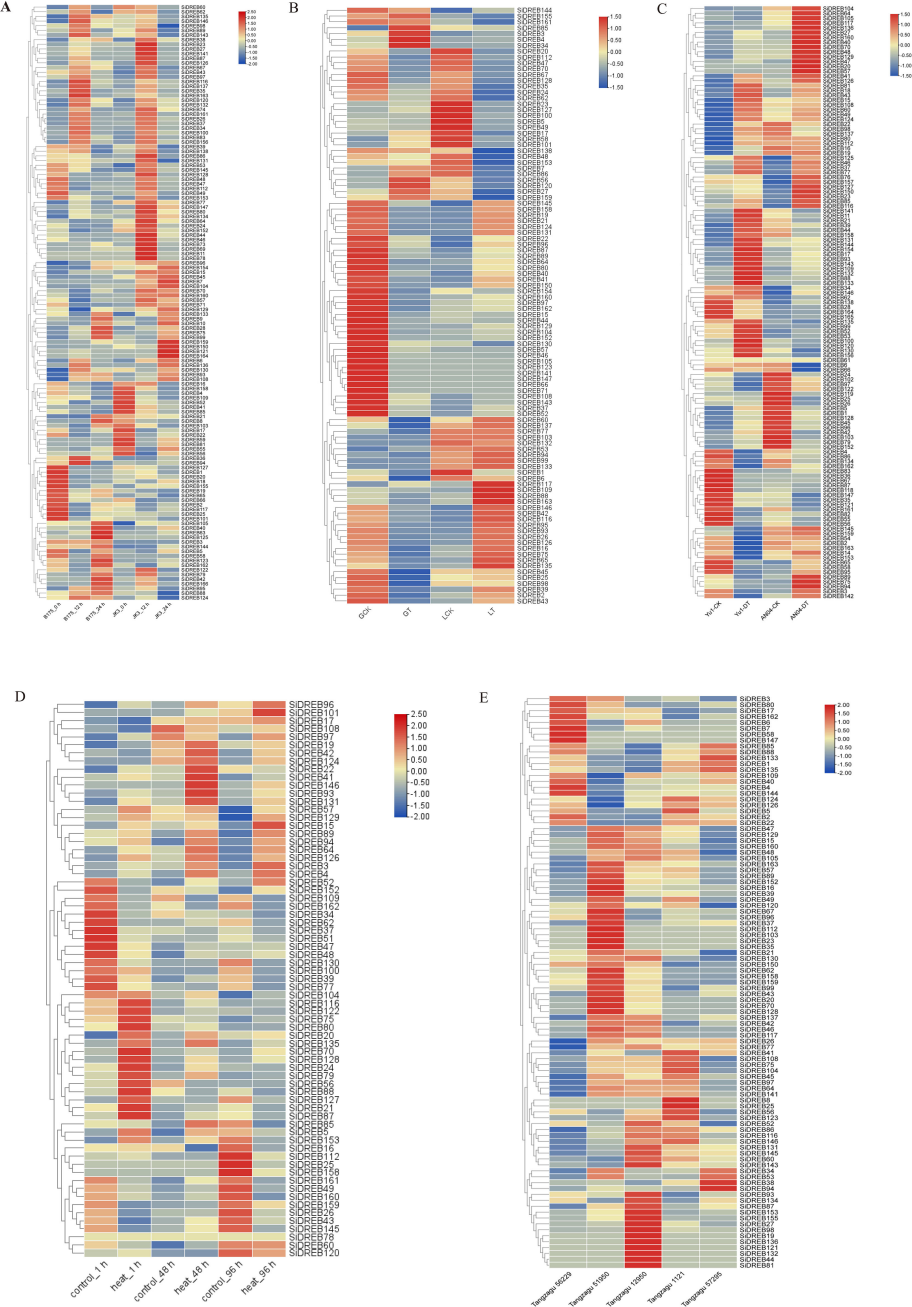
Expression analysis of *SiDREBs* under five abiotic stresses. **(A)** Expression analysis under saline-alkali stress. **(B)** Expression analysis under herbicide stress, in which GT and LG31 represent atrazine-resistant variety GA2 and atrazine-sensitive variety LG31, respectively, and GCK and LCK are control treatments. **(C)** Expression analysis under drought stress. Yu1-DT and AN04-DT represent drought-resistant variety ‘ Yugu1 ‘ and drought-sensitive variety AN04, respectively. Yu1-CK and AN04-CK were control treatments. **(D)** Expression analysis under high temperature stress. **(E)** Expression analysis under shade stress. The expression levels in the figure are average.

In order to study the role of *SiDREBs* in responses to abiotic stresses in foxtail millet, we carried out *SiDREB* expression analysis, and constructed the expression heat map of SiDREBs under different abiotic stresses. In order to verify the function of *SiDREBs* under saline-alkali stress, we used the gene expression data in B175 (susceptible) and JK3 (resistant) varieties obtained at 0, 12 and 24 h under saline-alkali stress to analyze the expression of *SiDREB* genes. The expression of a total of 128 *SiDREBs* was detected (**Figure 8A**, **Supplementary Table S3**). The expression patterns of these 128 genes at different time points were analyzed (**Supplementary Table S3**, **Supplementary Figure S4**). We further screened the DEGs in 12 h *vs* 0 h and 24 h *vs* 12 h in B175 and JK3, respectively (**Supplementary Table S3**). The results showed that there were 47 DEGs (16 up-regulated and 31 down-regulated) in B12 *vs* B0 comparison. In B24 *vs* B12 comparison, there were 30 DEGs (20 up-regulated and 10 down-regulated). There were 34 DEGs (20 up-regulated and 14 down-regulated) in J12 *vs* J0 comparison. In J24 *vs* J12 comparison, there were 38 DEGs (17 up-regulated and 21 down-regulated). Further analysis showed that *SiDREB153* was down-regulated in B12 *vs* B0 comparison while up-regulated in J12 *vs* J0 comparison, indicating that this gene is involved in positively regulating the response of JK3 to saline-alkali stress.

Atrazine residue in soil is a main abiotic stress of foxtail millet (Sun et al., 2022). In this study, based on the transcriptome data of known atrazine-resistant variety (Gongai2, GA2) and atrazine-sensitive variety (Longgu31, LG31), we analyzed the expression levels of *SiDREBs* under atrazine stress. A total of 101 expressed *SiDREB* genes were detected (**Figure 8B**, **Supplementary Table S4**). We then analyzed the DEGs (**Supplementary Table S4**). The results showed that compared with the control, there were 35 DEGs (three up-regulated and 32 down-regulated) in GA2 under herbicide stress. Compared with the control, LG31 under herbicide stress exhibited a total of 33 DEGs (12 up-regulated and 21 down-regulated), and *SiDREB15/44/70/128* were down-regulated after herbicide stress in both varieties. Since the herbicide-resistant variety GA2 had a larger number of down-regulated genes than the herbicide-sensitive variety LG31, it could be speculated that *SiDREBs* may play a negative regulatory role under herbicide stress. It is worth noting that *SiDREB56/58* was up-regulated in GA2 and down-regulated in LG31, indicating that these two genes participate in the herbicide stress process through positive regulation. Therefore, these two genes may be candidate genes for herbicide stress.

Foxtail millet has strong drought tolerance and is a model plant for studying stress biology. In this study, we analyzed the expression level of *SiDREBs* under drought stress based on the transcriptome data of drought resistant variety ‘Yugu1’ and drought-sensitive variety AN04. A total of 123 expressed *SiDREB* genes were detected (**Figure 8C**, **Supplementary Table S5**). We then analyzed the DEGs (**Supplementary Table S5**). The results showed that compared with the control, there were 33 DEGs (21 up-regulated and 12 down-regulated) in ‘Yugu1’ after drought stress, and 18 DEGs (13 up-regulated and five down-regulated) in AN04 after drought stress. The expression of *SiDREB55/161* and *SiDREB37/46/109/132* was respectively down-regulated and up-regulated after drought stress in both varieties. In general, it can be speculated that *SiDREBs* play a positive regulatory role in response to drought stress. Because the expression of *SiDREB132* is high under drought stress, it may be a candidate gene for drought stress.

High temperature has a negative impact on plant growth and development, thus threatening global agricultural security. In this study, based on the existing transcriptome data of foxtail millet leaves under high temperature, we analyzed the expression of *SiDREBs* under high temperature stress at different times (1, 48, and 96 h). A total of 68 expressed *SiDREB* genes were detected (**Figure 8D**, **Supplementary Table S6**). The DEGs were also analyzed (**Supplementary Table S6**). The results showed that compared with the control, there were 22 DEGs (13 up-regulated and nine down-regulated) after 1 h of high temperature stress. After 48 h of high temperature stress, there were 12 DEGs (eight up-regulated and four down-regulated). After 96 h of high temperature stress, there were 28 DEGs (13 up-regulated and 15 down-regulated). SiDREB79/93 were up-regulated after 1, 48, and 96 h of high temperature stress, indicating that these two genes play a positive regulatory role in response to high temperature stress. On the contrary, SiDREB37/109 were down-regulated at all the time points. Therefore, the up-regulated *SiDREB79/93* may be candidate genes for high temperature stress.

In order to explore the function of *SiDREBs* under shade stress, the transcriptome data of two shade-tolerant varieties (Tangzagu 56229 and Tangzagu 51950) and three shade-intolerant varieties (Tangzagu 12950, Tangzagu 1121, and Tangzagu 57295) under shade stress were used to analyze the expression of *SiDREBs*. A total of 97 expressed *SiDREB* genes were detected (**Figure 8E**, **Supplementary Table S7**). Differential expression analysis was performed between the shade-tolerant and shade-intolerant varieties (**Supplementary Table S7**). Tangzagu 56229 had 24 DEGs (three up-regulated and 21 down-regulated) relative to Tangzagu 12950, 21 DEGs (seven up-regulated and 14 down-regulated) relative to Tangzagu 1121, and 13 DEGs (eight up-regulated and five down-regulated) relative to Tangzagu 57295. *SiDREB7* and *SiDREB34/77* were respectively up-regulated and down-regulated in the three comparison groups. Tangzagu 51950 had 21 DEGs (11 up-regulated and 10 down-regulated) relative to Tangzagu 12950, 16 DEGs (11 up-regulated and five down-regulated) relative to Tangzagu 1121, and 24 DEGs (18 up-regulated and six down-regulated relative to Tangzagu 57295. *SiDREB16/37/67/152/158* were up-regulated and *SiDREB126/144* were down-regulated in the three comparison groups. Therefore, these up-regulated genes (*SiDREB7/16/37/67/152/158*) may be candidate genes for shade stress.

## 4 Discussion

### 4.1 Characterization of *SiDREBs* during evolution

Based on plant genome sequencing, the DREB gene family has been widely studied in various plants. In this study, we identified a total of 166 *SiDREBs* and analyzed their functions. The number of members in the DREB family varies significantly between greatly species. The number of *SiDREB* family members is greater than that in *Ananas comosus* (Agarwal et al., 2006) and *Glycine max* (Hou et al., 2022), but smaller than that in *Gossypium* (Su et al., 2023) and *Triticum aestivum* (Novillo et al., 2004). These differences may be related to the size of the genome (Buck and Atchley, 2003), and a similar conclusion has been reported in the study of Wang et al (Wang et al., 2025). Subcellular localization prediction revealed that SiDREBs are mainly localized in the nucleus, which is similar to the findings of Mushtaq et al (Mushtaq et al., 2021), confirming that DREB transcription factors mainly play an important role in regulating gene expression (Agarwal et al., 2006). The phylogenetic tree showed that six subfamilies could be distinguished among *SiDREBs*, which is consistent with the classification results in Arabidopsis. The number of members in each subfamily varies greatly among different species. For example, there are 118 members in the A2 subfamily of SiDREBs in this study, accounting for 71% of the total family members, but there are only eight members in the A2 subfamily of Arabidopsis (Sakuma et al., 2002), which only accounts for 14% of the total family members. The reason for this difference may be due to different genetic patterns of plants, which may lead to species specificity in the distribution pattern of members in different subfamilies.

Our results revealed that members in the same subfamily have similar motif structure, number, and distribution, but there were large differences between subfamilies, which leads to different functions of different subfamilies. Differences in motifs are not only related to the formation of branches in the phylogenetic tree, but also responsible for interaction with different protein molecules, which is conducive to DNA binding (Defoort et al., 2018; Maqsood et al., 2022). In addition, functional differences between members of different subfamilies may be due to structural differences between introns and exons, which can also drive the evolution of polygenic families (Rogozin et al., 2003). In this study, most SiDREB family members did not contain introns, and the number of introns varied from 1 to 9, which is consistent with previous studies in plants such as apple (Li et al., 2019), pear (Gong et al., 2019), and grape (Wang et al., 2020). It has been demonstrated that intronless genes play important roles in biological regulation and stress response (Jain et al., 2008). Therefore, it can be speculated that the small number of introns in *SiDREBs* may help to resist stress.

Generally, genome-wide replication events occur in two ways: tandem replication and fragment replication (Maqsood et al., 2022). These two forms of replication are main forces driving plant genome evolution, and can genetic system can be altered by creating new subfamilies (Cannon et al., 2004). In this study, repeated gene analysis of the *SiDREB* family revealed that there were 18 pairs of tandem repeat genes and 21 pairs of fragment repeat genes. Therefore, it can be speculated that fragment repeats and tandem repeats may be the main way for the amplification of the gene family, which is consistent with the results of the *Vitis vinifera* DREB gene family (*VvDREB*) reported by Wanye (Wan, 2023).

The expression pattern of genes are frequently highly correlated with their biological functions, and understanding the expression level of genes can help to infer their functions in plant growth (Ortiz-Lopez et al., 2000). This study found that 166 *SiDREBs* were expressed in leaves, roots, shoots, and panicles of foxtail millet, but the expression levels were different, and most of them were highly expressed in roots and leaves. These results are consistent with the highest expression of Arabidopsis DREB family genes in roots and leaves (Shen et al., 2003). The above results show that *SiDREBs* play important roles in the different growth and development processes of foxtail millet.

### 4.2 *SiDREBs* play an important role in abiotic stress response

By analyzing the *cis*-acting elements of the promoter of *SiDREBs*, gene function can be preliminarily predicted. We found that *SiDREBs* contain a variety of elements related to hormone and abiotic stress responses, and the same result is also studied in the *MnDREBs* of *MorusalbaL* (Wu et al., 2022), indicating that *SiDREBs* play a role in tolerance to adversity of foxtail millet.

DREB is widely involved in reacting to different kinds of stresses such as drought, salt, cold, and high temperature (Xu et al., 2011). In this study, KEGG enrichment results showed that *SiDREBs* were enriched in the MAPK signaling pathway, which has been found to play an important role in abiotic stress signal transduction (Danquah et al., 2014). Therefore, we further explored the response mechanism of *SiDREBs* to five abiotic stresses, including saline-alkali, high temperature, drought, shade, and herbicide. The results showed that the expression levels of most *SiDREBs* changed under some abiotic stresses. We found that *SiDREB153* is involved in the process of saline-alkali stress through positive regulation. Subsequently, we will clone *SiDREB153*, transform it into *Arabidopsis thaliana* and *Setaria italica*, and further study the molecular mechanism for its function by Y2H, Co-IP, EMSA, and ChIP. The expression levels of some genes increased under various stresses, such as *SiDREB80* under drought, saline-alkaline and shade stress, *SiDREB96* under drought and shade stress, *SiDREB135* under drought and high temperature stress, *SiDREB159* under herbicide and saline-alkaline stress. Similar findings have also been reported in other studies. For example, the expression of *DREB* genes in Arabidopsis increased under low temperature, shade, and other stress treatments (Novillo et al., 2004). In addition, the *GmDREB2A* transgenic soybean showed significantly enhanced drought tolerance (Mizoi et al., 2012). Under high temperature stress, the tolerance of *AtDREB2A* transgenic Arabidopsis to heat stress was significantly enhanced (Sakuma et al., 2006). Overexpression of *OsDREB2A* in rice enhanced its tolerance to salt stress without changing its total nutrients (Mallikarjuna et al., 2011). Overexpression of *MtDREB2A* significantly enhanced the tolerance of *Medicago sativa* to salt stress (Chen et al., 2009). Overexpression of *TaDREB3* improved the tolerance of *Triticum aestivum* to high temperature and salt stress (Niu et al., 2020). Therefore, it can be speculated that *SiDREB* genes can enhance the tolerance of foxtail millet to abiotic stress. In this study, the response of *SiDREBs* to abiotic stress was systematically described by analyzing their expression patterns of *SiDREBs* under five abiotic stresses. We found some important SiDREBs that positively (*SiDREB56/58/153*) or negatively (*SiDREB37/109/126/144*) regulate abiotic stress response in foxtail millet. *SiDREBs* were found to be responsive to various abiotic stresses (*SiDREB80/159*). This study lays an important theoretical foundation for further research on *SiDREB* gene function.

## 5 Conclusion

In this study, a total of 166 *SiDREBs* were identified, which were divided into six subfamilies and were unevenly distributed on nine chromosomes. Multiple SiDREBs were localized in the nucleus. *SiDREBs* have tissue-specific expression, with most genes being highly expressed in roots and leaves. *SiDREBs* contain multiple stress responsive elements. Most *SiDREBs* are up-regulated under abiotic stress, and these genes work together to play an important role in abiotic stress response, particularly *SiDREB153*, which was found to play a critical positive regulatory role in the response of foxtail millet to saline-alkali stress.

## Data Availability

The datasets generated and analyzed during the current study are available in the Gene Expression Omnibus database (https://www.ncbi.nlm.nih.gov/geo/) (accession number: GSE278652).

## Supplementary material

Supplementary Figure 1 Conserved motifs and gene structures of *SiDREBs*. **(A)** Conserved motifs in the 166 SiDREBs. Different colored blocks represent different motifs. **(B)** Gene structures of *SiDREBs*. Consisting of CDS (red), UTR (green), and introns (black lines).

Supplementary Figure 2 The secondary structure analysis of SiDREBs protein

Supplementary Figure 3 The tertiary structure analysis of SiDREBs protein

Supplementary Figure 4 Co-expression trend analysis of *SiDREBs.* **(A)** The expression trend of B175, **(B)** The expression trend of JK3. The gray line represents the genes under the same expression trend, and the blue line represents the average level.
