# Supplementary figures and images for: Identification of DREB gene family in foxtail millet (Setaria italica) and analysis of its expression pattern in response to abiotic stress
Source: Front Plant Sci. 2025 Apr 28;16:1552120. doi: 10.3389/fpls.2025.1552120 (PMC12066435; doi:10.3389/fpls.2025.1552120)

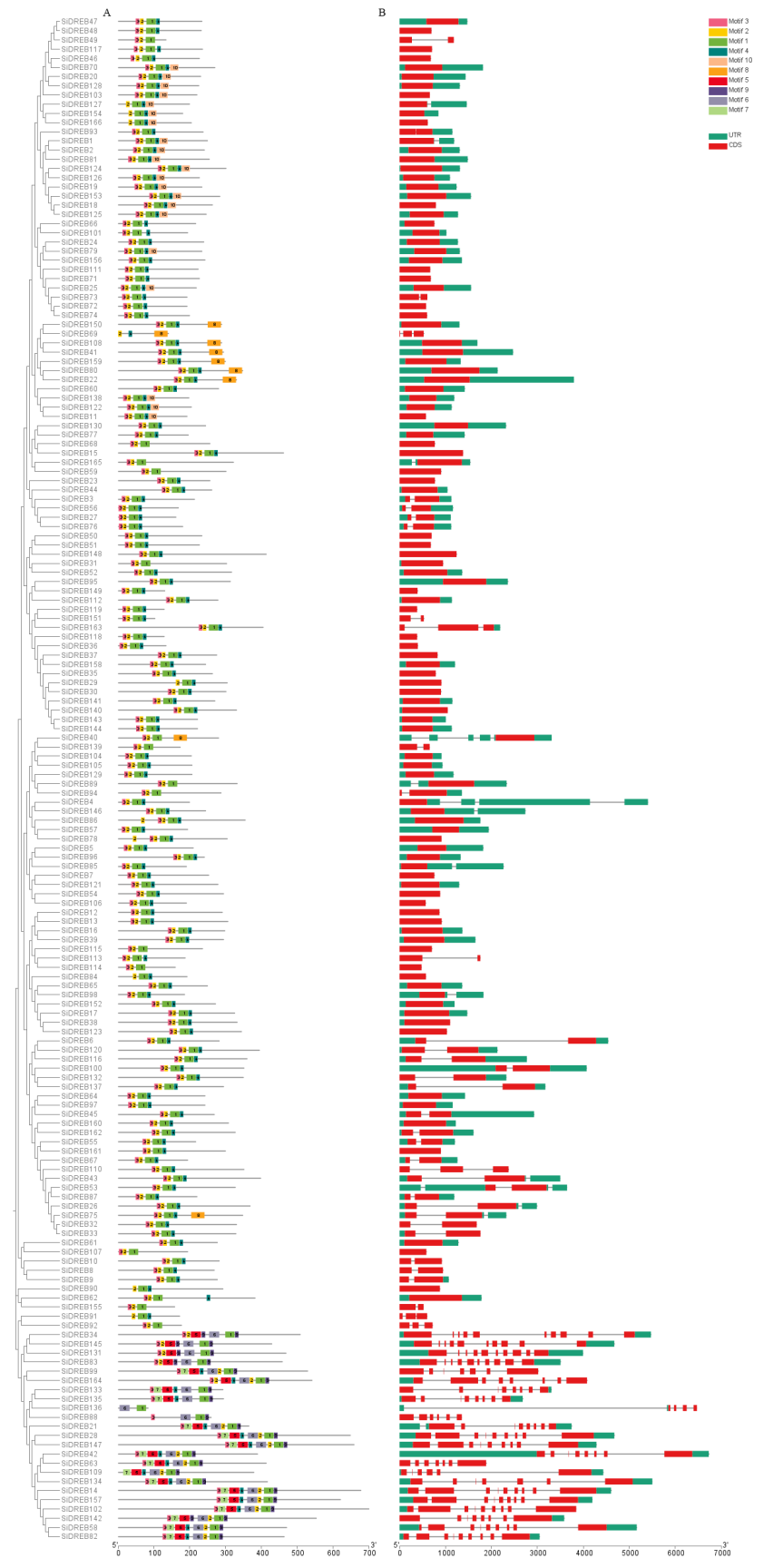

Supplement: Supplementary Figure 1 — Conserved motifs and gene structures of SiDREBs. (A) Conserved motifs in the 166 SiDREBs. Different colored blocks represent different motifs. (B) Gene structures of SiDREBs. Consisting of CDS (red), UTR (green), and introns (black lines). [file Image1.tif]

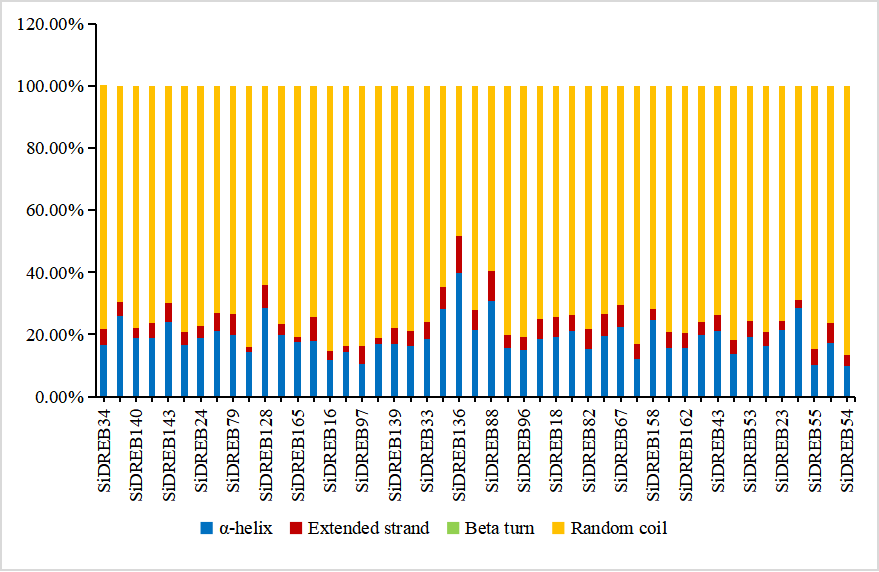

Supplement: Supplementary Figure 2 — The secondary structure analysis of SiDREBs protein [file Image2.png]

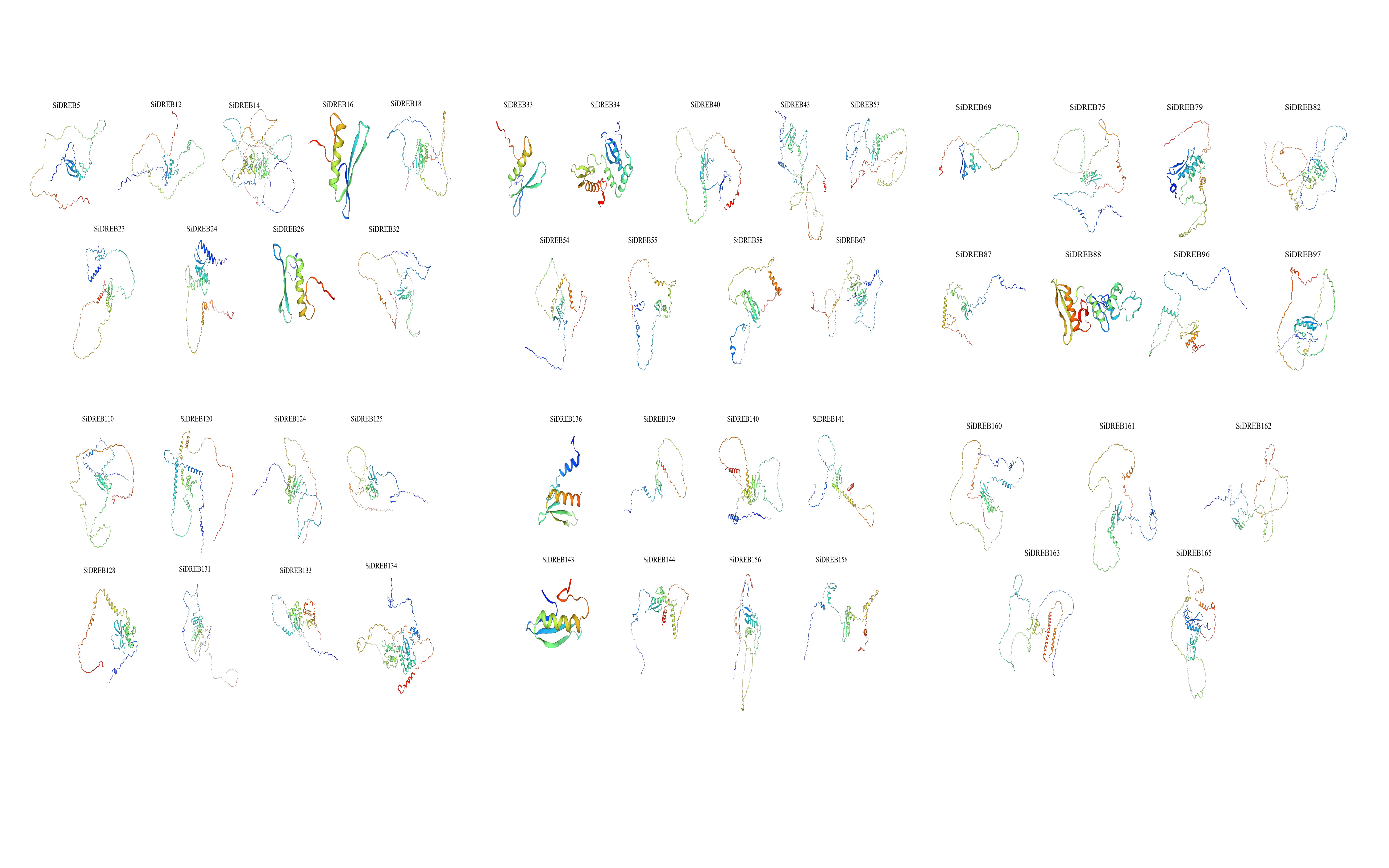

Supplement: Supplementary Figure 3 — The tertiary structure analysis of SiDREBs protein [file Image3.jpeg]

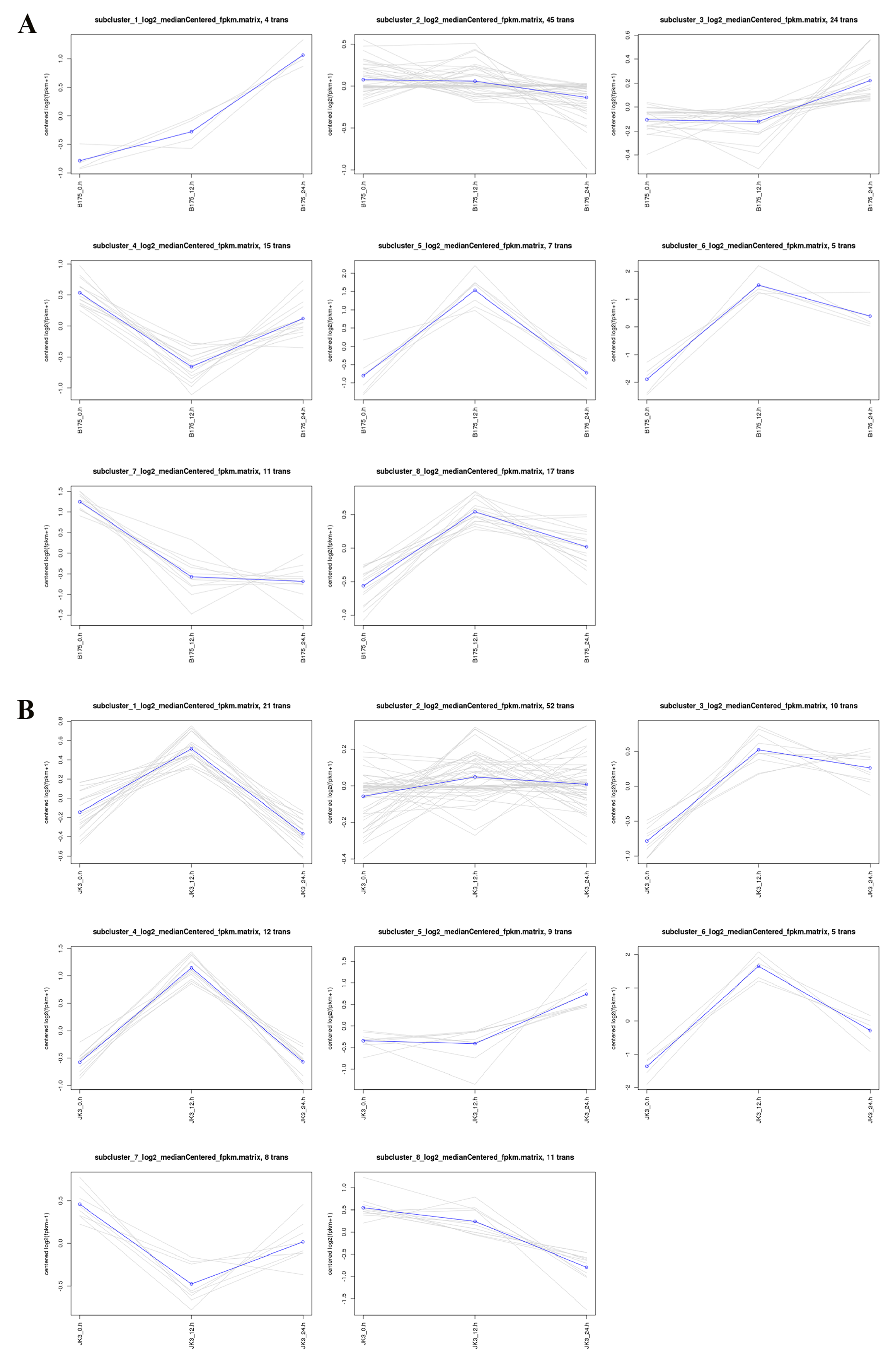

Supplement: Supplementary Figure 4 — Co-expression trend analysis of SiDREBs. (A) The expression trend of B175, (B) The expression trend of JK3. The gray line represents the genes under the same expression trend, and the blue line represents the average level. [file Image4.tif]
